# Fiscal Incidence in Ghana

**DOI:** 10.1111/rode.12299

**Published:** 2017-01-11

**Authors:** Stephen D. Younger, Eric Osei‐Assibey, Felix Oppong

**Affiliations:** ^1^ Department of Economics Ithaca College Ithaca NY 14850 USA; ^2^ Department of Economics University of Ghana Legon Ghana; ^3^ World Bank 8th Avenue Extensioin Accra Ghana

## Abstract

We use methods developed by the Commitment to Equity Institute to assess the effects of government taxation, social spending and indirect subsidies on poverty and inequality in Ghana. We also simulate several policy reforms to assess their distributional consequences. Results show that, although the country has some very progressive taxes and well‐targeted expenditures, the extent of fiscal redistribution is small, but about what one would expect given Ghana's income level and relatively low initial inequality. Results for poverty reduction are less encouraging: were it not for the in‐kind benefits from health and education spending, the overall effect of government spending and taxation would actually increase poverty in Ghana. Eliminating energy subsidies and at the same time reallocating part of the savings to well‐targeted transfer programs could lower the fiscal deficit while reducing inequality and protecting the poor.

## 1 Introduction

One of the functions of government is to redistribute resources, especially to the most disadvantaged members of society. Although there is considerable disagreement over both the extent and the means to effect such redistribution, most people agree that society is better off if inequality and poverty can be reduced, and all governments do, in fact, redistribute income with their tax and expenditure policies, though not always progressively. The purpose of this paper is to examine the extent to which the government of Ghana does so. In particular, the paper addresses three general questions:

1. How much redistribution and income poverty reduction is being accomplished through social spending, subsidies and taxes?
2. How progressive are revenue collection, subsidies and government social spending?
3. Within the limits of fiscal prudence, what could be done to increase redistribution and poverty reduction through changes in taxation and spending?

Ghana is an interesting case for such analysis for several reasons. First, the government of Ghana regularly commits itself to reducing poverty and inequality and increasingly adopts policies explicitly intended to alter the distribution of income. Examples include a conditional cash transfer (the Livelihood Empowerment against Poverty program); elimination of school fees; free school meals in educationally deprived districts; and targeted fee exemptions for the National Health Insurance Scheme (NHIS). While such policies are common in middle‐income countries, African governments are only beginning to consider them. Ghana's results can thus provide some guidance for other African policymakers.

Second, Ghana's taxation and public spending have increased as a share of gross domestic product (GDP) in recent years. That combined with the increase in targeted transfers and increased coverage for both education and health services might lead one to believe that Ghana is redistributing a larger share of national income. The study estimates the overall effect of government spending and taxation—the “fisc”—on the distribution of income. As we will see, despite the increased size of the budget and great concern for distributional issues in the country, this effect is rather small and is only about what one would expect for a country of Ghana's GDP per capita and overall inequality.

## 2 Methods and Approach

**Figure 1:**
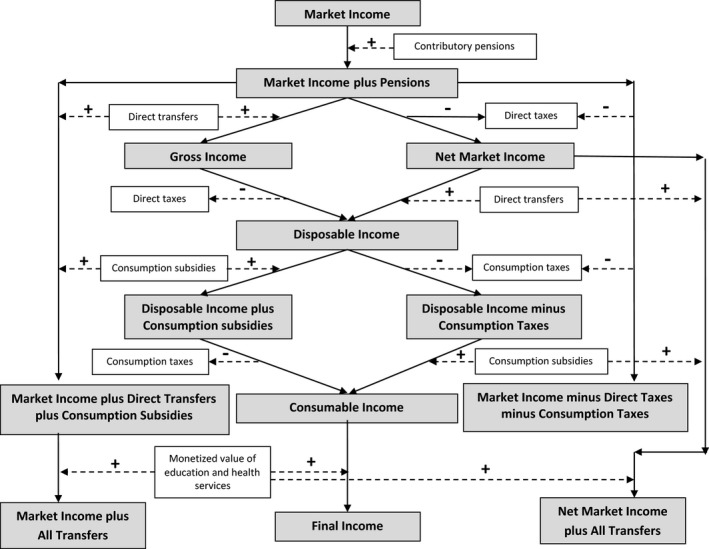
*Definition of CEQ Income Concepts* *Source:* Lustig and Higgins (2017).

The paper uses incidence analysis, a description of who benefits when the government spends money and who loses when the government taxes, following the methods developed by the Commitment to Equity (CEQ) Institute^1^ (Lustig, 2017). Although it is possible to use incidence analysis to examine one particular expenditure or tax, the thrust of the CEQ analysis is rather to get a comprehensive picture of the redistributive effect of as many tax and expenditure items as possible. This is accomplished by comparing standard poverty and inequality measures^2^ for five core income concepts and eight complementary ones. Figure 1 shows the relationship between these income measures and helps to illustrate how we use them to analyze the distributional effects of fiscal policy.

“Market income” is income before the government has any influence on the income distribution with its tax and spending policies. It includes all earned and unearned income, except government transfers and contributory pension receipts. There is some debate as to whether pensions should be considered as deferred compensation for previous employment, and thus earned income, or a transfer payment. For Ghana, where almost all retirement benefits are either contributory (via the Social Security and National Insurance Trust [SSNIT]) or for former public sector employees (e.g. the CAP 30 pension scheme), it is best to view pensions as deferred compensation (see SSNIT, 2013). SSNIT does not receive any subsidy from the central government and makes pension payments that are actuarially fair. As such, the payout that SSNIT beneficiaries receive has no component of transfer from the central budget. The older pension schemes did not involve explicit withholding during the working years and are funded out of the central budget, but they are entirely for former public sector employees. Therefore, the pension is best viewed as a type of deferred compensation, part of the package of benefits, current and future, that a public employee receives. For these reasons, we take “market income plus pensions” as the “pre‐fisc” income concept for Ghana.

“Disposable income” is cash income available after government has taken away direct taxes such as personal income tax and distributed direct transfers such as Livelihood Enhancement Against Poverty (LEAP), as well as “near cash” transfers such as free meals at school. Because these two instruments often have very different distributional consequences, it is sometimes helpful to consider their influence separately, thus the two intermediate income concepts between market and disposable income in Figure 1. “Gross income” is market income plus direct transfers; “net market income” is market income less direct taxes.

While that is the end of government's impact on nominal cash income, many government policies affect households’ real income indirectly by altering the prices they pay. “Consumable income” is disposable income less indirect taxes—VAT, import duties and excise taxes—plus indirect subsidies, such as the support that government gives to electricity generators and distributors. Again, there are two intermediate income concepts between disposable and consumable income to capture the effect of indirect taxes and subsidies separately.

The last way that government influences the income distribution is through the provision of free or subsidized services such as health and education. “Final income” is consumable income plus the value of these in‐kind benefits, less any user fees paid for those services. Moving from consumable to final income highlights the effect on poverty and inequality of public health and education expenditures.

Our assumptions on the economic incidence of taxes are simple: direct taxes are born entirely by the income earner; indirect taxes are born entirely by the consumer. This latter assumption is not entirely appropriate if markets are not competitive and many are not in Ghana. However, the extent to which monopolies or oligopolies shift indirect taxes to consumers is not clear and could be either greater or less than 100%, depending on the functional form of the demand function (Fullerton and Metcalf, 2002). Since we have no information on those functional forms, we assume that 100% of taxes are shifted to consumers regardless of market structure, but we also check the sensitivity of our results by assuming that only 50% of all indirect taxes are shifted to consumers.

The one exception we have made to these simple incidence assumptions is the fertilizer subsidy, which we assume falls on the food producers that receive it, not food consumers.

## 3 Data

To understand the distributional consequences of taxes and public expenditures, we need data on all of the above income concepts for a representative sample of individuals in the country. We construct income distributions for each income concept outlined in the previous section and derive summary statistics for those distributions using the 2012/13 Ghana Living Standards Survey (GLSS), round 6 (see GSS, 2014). In addition, we use administrative tax and expenditure data from fiscal year 2013 to estimate some of the information needed, most specifically, the per beneficiary amount of spending on public education and health services.^3^

### Consistency between Administrative and Survey Data Sources

It is possible to calculate the total amount that the government spends on certain items and taxes on others using both administrative data—the national accounts, the budget, etc.—and data from the GLSS survey. Because the GLSS is a nationally representative survey, these amounts should coincide, but they sometimes do not. This can lead to errors in our estimate of distributional effects if the degree of inconsistency varies among the tax, expenditure and income variables used in the analysis.

In Ghana, the GLSS estimates and the public accounts are quite consistent for most of the items we consider in this study. Only our original estimates of both the benefit associated with public in‐patient health care and the number of in‐patients at public facilities was far too high, so we have scaled that benefit down by a factor of 0.33 so that our estimate of total benefits in the survey matches the administrative accounts.^4^

### Description of Taxes and Expenditures in Ghana

Table 1 gives the breakdown of the major government revenue sources in 2013, the fiscal year that coincides most closely with the GLSS survey that ran from October 2012 to October 2013. Overall revenues are small as a share of GDP, only 21%, a fact that limits government's ability to affect the distribution of income. Revenues are rather balanced between direct taxes, indirect taxes and non‐tax revenues.^5^ VAT (including the National Health Insurance Levy (NHIL)) is the most important single source, followed by the corporate income tax, personal income tax and import duties.

**Table 1:** Government Revenues, Ghana, 2013, Million Cedis

	Amount (millions)	Comparable GLSS‐6 estimate	Share of total government revenue (%)	Share of GDP (%)	Included in CEQ analysis?
*Total revenue*	19,472			20.83	
*Taxes*	14,467		74.3	15.48	
*Direct taxes*	6,302		32.4	6.74	
Personal income tax	2,549	2,635	13.1	2.73	yes
Corporate income tax	2,734		14.0	2.93	no
Other direct taxes	1,018		5.2	1.09	no
*Indirect taxes*	7,312		37.5	7.82	
Vat	3,317	1,891	17.0	3.55	yes
Nhil	648	630	3.3	0.69	yes
Import duties (less exemptions)	2,231	1,059	11.5	2.39	yes
Cocoa export duties	100	39	0.5	0.11	yes
*Excises*	868		4.5	0.93	
Petroleum excises	525	593	2.7	0.56	yes
Communications services tax	174	119	0.9	0.19	yes
Other excises	169	124	0.9	0.18	yes
Other indirect taxes	148		0.8	0.16	no
*Other taxes*	1,368		7.0	1.46	
of which SSNIT contributions	1,048	1,953	5.4	1.12	yes
non‐pension SSNIT contributions (NHIL)	159		0.08	0.17	yes
*Non‐tax revenues*	5,005		25.7	5.35	
Internally generated funds	2,516		12.9	2.69	yes
Other non‐tax revenues	2,489		12.8	2.66	No
Note: Share of government revenue included in analysis:			69.0		
Note: Share of GDP included in analysis:				14.4	

Overall, our analysis treats tax items that account for 69% of total government revenues and 14.4% of GDP. The most significant items we cannot cover are corporate income tax and non‐tax revenues other than internally generated funds.

It is much more difficult to attribute the expenditure side of the budget to specific beneficiaries. Governments spend significant amounts of their budgets on genuine public goods: national defense, law enforcement and public administration. By their nature, these goods and services are not attributable to individuals. The areas in which we can identify specific beneficiaries are usually social expenditures: transfer payments, health and education. Table 2 gives a breakdown of expenditures in Ghana in 2013. Overall, we can analyze only 34.2% of total expenditures in our analysis, amounting to 9.8% of GDP. Education spending is by far the largest part of social spending in the analysis, followed by health spending and pensions. But notice that indirect subsidies, especially for electricity, are almost as large as pensions.

**Table 2:** Government Expenditures, Ghana, 2013, Million Cedis

	Amount (millions)	Comparable GLSS‐6 estimate	Share of total government spending (%)	Share of GDP (%)	Included in CEQ analysis?
Total government spending, including SSNIT pensions	26,729			28.60	
Primary government spending	22,332		83.5		
Social spending	6,906				
*Direct transfers* a	70		0.3	0.07	
LEAP	18	1	0.1	0.02	yes
School feeding program^b^	52	61	0.2	0.06	yes
*Total in‐kind transfers*	6,893		25.8	7.38	
*Education* c	5,282		19.8	5.65	
Pre‐school	147	122	0.6	0.16	yes
Primary	1,243	1,270	4.6	1.33	yes
Junior high school	532	534			yes
Senior high school	546	629	2.0	0.58	yes
Vocational	34	45	0.1	0.04	yes
Teacher training	96	104	0.4	0.10	yes
Nursing school	319	196	1.2	0.34	yes
Polytechnic	121	128	0.5	0.13	yes
University	520	1,261	1.9	0.56	yes
Other education spending	1,724		6.5	1.84	no
*Health*	1,555	1,916	5.8	1.66	
Contributory^d^	159		0.6	0.17	yes
Noncontributory	1,396		5.2	1.49	yes
Inpatient services		625			yes
Outpatient services		1,291			yes
Housing and urban	56		0.2	0.06	no
*Contributory pensions*	1,234		4.6	1.32	
SSNIT pensions^e^	443	201	1.7	0.47	yes
Other pensions, gratuities, and end‐of‐service benefits	791	264	3.0	0.85	yes
Non‐social spending					
*Indirect subsidies*	1,231		4.6	1.32	yes
On final goods (electricity lifeline tariffs)	1		0.0	0.0	yes
On inputs (electricity and petroleum products)	1,158	1,268	4.3	1.24	yes
On fertilizer	72	58	0.3	0.08	yes
*Other primary spending*	11,692		43.7	12.51	no
*Debt servicing*	5,609		21.0	6.00	no
Interest payments	4,397		16.5	4.70	no
Amortization payments	1,212		4.5	1.30	no
Note: Share of government spending included in analysis:			34.4		
Note: Share of GDP included in analysis:				9.8	

Notes

^a^There are other quasi‐cash transfers such as school uniforms, but we have no information on their budget, nor on the total budget for direct transfers. ^b^This estimate comes from information on school feeding expenditures in January–April of 2014, not 2013. ^c^Education and health do not include spending of internally generated funds. ^d^This comprises SSNIT contributions to the NHIS on behalf of SSNIT members. ^e^SSNIT pensions are not usually consolidated into the central government accounts, as it is an independent institution. *Sources:* Ministry of Finance and Economic Planning, Controller and Accountant General's Department, SSNIT, Ministry of Education, and authors’ calculations. For spending on the Ghana School Feeding Program, communication from the directorate. For spending on fertilizer subsidies, communication from the Ministry of Agriculture.

## 4 Results^6^

### Inequality and Poverty

Table 3 gives the Gini coefficients and headcount indices for both local and international, purchasing power parity (PPP)‐based poverty lines for each CEQ income concept.^7^ Considering inequality first, Table 3 shows that there are only two places in the transition from market income to final income where government taxation and spending have a noticeable effect on the Gini coefficient: from gross income (or market income) to net market income and from consumable to final income. The transition from gross to net market income reflects the imposition of direct taxes on persons; in Ghana, this is only pay as you earn (PAYE) and presumptive taxation of small businesses. In the next section, we will see that these are progressive taxes, falling disproportionately on high‐income earners. It is also one of the larger sources of tax revenue in Ghana. These two features mean that direct taxes^8^ have a small but statistically significant effect on the Gini coefficient of about 0.012.

**Table 3:** Gini Coefficients and Poverty Indices for CEQ Income Concepts

	Poverty line:	GH₵1314 per year		GH₵792 per year	US$1.25 per day at PPP	US$2.50 per day at PPP	US$4.00 per day at PPP
	Gini	Headcount index	Poverty gap	Headcount index	Headcount index	Headcount index	Headcount index
Market income + Pensions	0.437	0.240	0.078	0.083	0.060	0.264	0.489
Market income	0.438	0.243	0.080	0.086	0.062	0.267	0.493
Gross income	0.436	0.238	0.076	0.081	0.058	0.262	0.488
Net market income	0.425	0.244	0.079	0.086	0.061	0.269	0.500
Disposable income	0.424	0.242	0.078	0.084	0.059	0.268	0.499
Disposable income + Indirect subsidies	0.424	0.235	0.075	0.080	0.057	0.258	0.489
Disposable income – Indirect taxes	0.423	0.272	0.089	0.100	0.070	0.298	0.536
Consumable income	0.423	0.262	0.086	0.094	0.068	0.288	0.522
Consumable income + In‐kind education	0.409	0.201	0.057	0.053	0.220	0.163	0.394
Final income	0.402	0.186	0.051	0.046	0.031	0.205	0.451

Notes

Data in the columns with US$ poverty lines at PPP are for per capita incomes to be comparable to other CEQ analyses; those in the columns with cedi poverty lines are per adult equivalent to be comparable to GSS publications. The national poverty line is GH₵1,314 (US$1,173 at PPP) per adult equivalent per year. The extreme poverty line is GH₵792.05 (US$707 at PPP) per adult equivalent per year. *Source:* GLSS‐6 and authors’ calculations.

The transition from consumable to final income shows the distributional impact of subsidized in‐kind health and education services. These are used rather equally across the income distribution and, like direct taxes, they are large shares of the budget. As such, they also reduce the Gini coefficient by a significant amount, 0.021. The lightly shaded row above final income shows consumable income plus education benefits only. One can see that two‐thirds of the reduction in the Gini from consumable to final income comes from education benefits, the rest from health.

Overall, the effect of the fisc on income distribution in Ghana is quite limited, reducing the Gini by 0.035 or about 8.6%. By comparison, Lustig (2015a) gives results for 11 middle‐income countries in Latin America that range from a reduction of 0.024 in Guatemala to 0.140 in Brazil, averaging 0.076. Lustig (2015b) includes four non‐Latin American countries—Armenia, Ethiopia, Indonesia and South Africa—with average reduction in the Gini of 0.035, exactly the same as Ghana, ranging from 0.003 in Ethiopia to 0.099 in South Africa. Regressing the reduction in the Gini from market to final income on GDP per capita at PPP and the initial inequality of market income for this (small) set of countries indicates that Ghana does about as well as one would expect, given its relatively low GDP per capita and initial inequality.

The effects of the fisc on poverty in Ghana are similar, with one exception. Comparing market income with disposable income, we see that pensions, direct taxes (PAYE), and cash transfers (LEAP and school feeding) have almost no effect on poverty.^9^ This is because neither pensions nor direct taxes affect the poor in Ghana; they are not in the formal economy or even the informal economy that pays presumptive tax. LEAP does benefit the poor, obviously, as does the school‐feeding program, but these are such small programs that their overall effect is miniscule.

Indirect subsidies for electricity (especially) and fertilizer reduce poverty by a small amount, about 1 percentage point at the higher poverty lines, but only 0.2 percentage points at the lowest poverty line. This reflects the fact that households with incomes in the US$1.25–$4.00 per day range do purchase electricity and thus benefit from its subsidy.

Looking at the transition from disposable income to disposable income less indirect taxes, we find one of the more unfortunate results that shows up consistently in CEQ analyses: indirect taxes increase poverty significantly. This is because taxes, even progressive ones that reduce inequality, cannot reduce poverty. Even the poorest households buy goods and services that pay VAT, import duties and excises. Nevertheless, notice that this effect is larger at the higher poverty lines: only 1.1 percentage points at the lowest poverty line in the table (US$1.25) but 3.7 percentage points at the highest (US$4.00), reflecting increasing consumption of taxable goods and services as incomes increase.^10^

The last transition, from consumable to final income, is much more encouraging. Here, we see substantial reductions in poverty. Because education and health expenditures are a large part of the budget and because they are relatively progressive, they are particularly helpful to poorer households in Ghana.

Overall, the fisc reduces poverty by 2.9 percentage points at the lowest poverty line and 3.8 at the highest. At the national poverty line, the fisc reduces poverty by 5.4 percentage points. Lustig (2015a) reports the change in the headcount ratio at the US$2.50 per day poverty line for 11 Latin American countries from market income (plus pensions) to consumable income (not final). These range from a reduction of 3.8% in Ecuador to an increase of 1.2% in Brazil and average a small reduction of 0.8%. Table 3 shows that a similar calculation for Ghana is an increase of 2.2%, greater than any of the countries that Lustig reviews. This highlights again the importance of in‐kind benefits from education and health services in Ghana's poverty reduction efforts: without them, the net effect of the fisc would be to increase poverty.

### Tax and Expenditure Incidence

**Figure 2:**
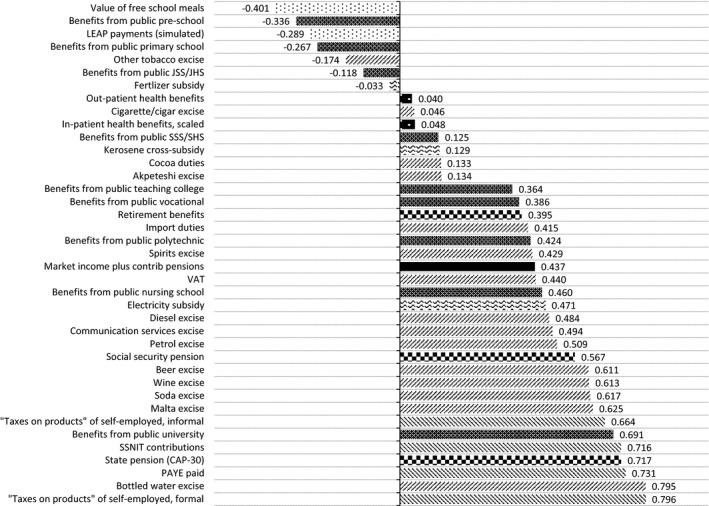
*Concentration Coefficients over Market Income plus Pensions* *Source:* GLSS‐6 (see GSS, 2014) and authors’ calculations.

A tax or expenditure has a larger distributional impact if it is strongly targeted to the poor or the rich and if it is large relative to incomes.^11^ In Ghana, Table 1 and Table 2 show how large each tax and expenditure item is relative to the budget and to GDP. Thus, we might expect that education expenditures or VAT may have large distributional consequences because they represent a large share of the budget and of GDP, but we also need to know how the benefits and costs of those items are distributed across the population—their incidence. Large taxes or expenditures that are distributed similarly to income will have little influence on the income distribution. To that end, Figure 2 shows concentration coefficients for the tax and expenditure items that we analyze in this paper.

LEAP is Ghana's only cash transfer program that explicitly targets the poor. It has a negative concentration coefficient of –0.289, as one would expect, but its concentration coefficient is somewhat smaller than that for similar cash transfer programs in middle‐income countries that also use a proxy means test for targeting, where they are closer to –0.4. We should keep in mind that our estimate is a simulation of beneficiaries based only on the eligibility criteria for individuals (elderly, vulnerable child, etc.) and the proxy means test (PMT) scores of GLSS‐6 households using the old PMT formula, which was in effect during the GLSS‐6 survey period. The LEAP program has another targeting mechanism that we cannot simulate, namely, community participation in the selection of recipients. That said, our estimate is close to a previous study that used a survey of actual LEAP recipients, along with GLSS‐5, and found a concentration coefficient of –0.33 (Tsimpo and Wodon, 2012).

There seems to have been some concern about the original targeting mechanism within the Ministry of Gender, Children and Social Protection, because the PMT formula has been recalculated using GLSS‐6 data. If we apply the revised formula rather than the prior one, the concentration coefficient improves considerably, to –0.65, though this recalculation has the advantage of being optimized to identify low incomes for the same dataset we are using, GLSS‐6.

The expenditure that is best targeted to the poor is the free school‐feeding program for selected primary and junior secondary schools, with a concentration coefficient of –0.401. This program has expanded significantly since 2009 and apparently to good effect. A previous study of school meals based on GLSS‐5 found a concentration coefficient of 0.126 (Joseph and Wodon, 2012).

Public spending on pre‐primary, primary and junior high school is also quite progressive, more so than is typical in middle‐income countries. One might think that this result reflects the fact that we have used per capita income as a welfare measure, but these concentration coefficients change relatively little if we use the Ghana Statistical Service (GSS) adult equivalence scale instead. The more plausible explanation is that richer households are more likely to choose private schools so that the benefits of public schools are more concentrated among the poor. Ghana has had excellent success expanding enrolment in pre‐primary and primary schooling. These concentration coefficients show that this has been a progressive change.

Higher levels of education are less progressive, though senior high school has a lower concentration coefficient than one might expect (0.125) given that relatively few students continue their studies to this level in Ghana. Benefits from teacher training and vocational schools are concentrated among better‐off households, though somewhat less so than income itself. Nursing school and polytechnic education are distributed about as (un)equally as income, while university education is far more concentrated among the rich than is income.

These patterns are similar to those found in many countries and, indeed, to Ghana's own past. They support the argument that, on equity grounds, it is better to subsidize lower levels of schooling than higher ones.

Ghana has also expanded access to public health facilities, not least through the creation of the National Health Insurance Scheme (NHIS), which provides free access to poor households. The concentration coefficients for inpatient and outpatient services at public facilities are close to zero, as they should be for a health system that provides access to the entire population. As with education, though, we might expect that richer households would opt for private health care providers, making the use of public facilities more progressive than we see here.

The three indirect subsidies in Ghana for fertilizer, kerosene and electricity have markedly different incidences. The fertilizer subsidy is equally distributed across incomes, with a concentration coefficient near zero. Kerosene has a slightly positive concentration coefficient, indicating that kerosene consumption rises with income, but its expenditure share declines with income. This is somewhat surprising, given that the rationale for reversing taxes on kerosene came from earlier incidence studies that found it to be a regressive tax (which it was).

The subsidy for electricity is actually regressive; electricity consumption is more concentrated among richer households than poorer ones. This is true even though we have taken into account the lifeline tariff structure in our analysis. The most likely explanation is that many of the poor do not have access to electricity connections. This subsidy was quite large in 2012 and 2013 and government has moved to eliminate it as part of its efforts to reduce the budget deficit. Our analysis suggests that this is a good decision on equity grounds, though as we noted in the previous section, it will affect some poor households, primarily in urban areas. In the following section, we consider this effect in detail, along with proposals to mitigate it.^12^

The last broad class of public expenditures in our analysis is pensions: social security (SSNIT), legacy pensions like CAP 30, and “retirement benefits,” which are probably some combination of end‐of‐service benefits and other retirement schemes outside the public sector. Both social security and pension benefits are collected disproportionately by richer households, which is to be expected, since these benefits are garnered only by former formal sector workers.

On the tax side, personal income taxes (PAYE) are very progressive in Ghana, in part because Ghana has a progressive rate structure, but also because the taxes fall on formal sector employees only. Social security contributions are not a tax *per se*, but their distribution is similar to that of PAYE. Both of these results are typical of other countries.

Taxes on the self‐employed are also highly progressive. This is not too surprising for those whose enterprise is formal (coefficient of 0.796), but it is also true of those who report that their enterprise is informal (coefficient of 0.664).

An unusual result is the progressivity of a large number of indirect taxes. VAT is neutral and import duties are slightly regressive, as is often the case for indirect taxes. Several of the excise taxes, however, are quite progressive, including those on many beverages (bottled water, soda, non‐alcoholic malted drinks, beer and wine); petroleum products (diesel and gasoline, but not kerosene); and communications services. As noted above, our estimate of the impact of petroleum duties includes indirect effects on the prices of goods and services that use petroleum as an input. Despite that estimation, petroleum taxes remain progressive.

There are also some quite regressive indirect taxes, including those on cigarettes and cigars, other tobacco products, the cocoa export duty and the excise tax on akpeteshie, a local spirit. For tobacco products and akpeteshie, government thus faces a trade‐off. It presumably would like to reduce consumption of these products for public health reasons, which argues for higher taxes, but those taxes will fall more heavily on poorer households than other taxes in the system. For the cocoa duty, however, there is no such dilemma. On both equity and efficiency grounds, there is no reason for this tax.

### How Can Ghana Do Better? Policy Simulations

Assuming that the government of Ghana would like its taxation and social expenditure policies to be more redistributive, what can it do? This section simulates several policy changes and analyzes their impact on inequality and poverty.

#### Subsidies to electricity and fuel

We start with the most topical policies: the government has announced that it will no longer subsidize electricity and fuel. These are significant changes in fiscal policy. In 2013, the year of this study, the government spent 1.1 billion cedis (1.24% of GDP) on electricity subsidies and indirectly subsidized fuel imports by offering the bulk oil companies an artificially low exchange rate, saving them about 600 million cedis in 2013. These are also controversial policies, eliciting significant public protest and a principal complaint is that eliminating these subsidies will harm the poor.

Table 4 shows that the share of electricity and fuel subsidies captured by the poor are small, as one would expect given their concentration coefficients. Still, eliminating these subsidies will harm some (few) poor people. How much is the damage? Table 5 gives the results for poverty and inequality measures of three CEQ income concepts for four simulations for elimination of electricity subsidies. The first simulation simply removes the subsidy. This increases the headcount by a little less than one percentage point at the national poverty line and less than half that at the extreme poverty line. These are small, but statistically significant increases in poverty. The compensation for government is a budget savings of 1.4% of GDP.^13^

**Table 4:** Shares of Population, Electricity, and Fuel Subsidies by Poverty Status

	Electricity	Fuel	Population
Extreme poor	0.008	0.011	0.084
Poor	0.041	0.042	0.158
Not poor	0.951	0.947	0.178

**Table 5:** Simulation Results for Eliminating Electricity Subsidies

Change in:		Simulation			
		(1)	(2)	(3)	(4)
Extreme poverty headcount	Disposable income			−0.013	−0.007
	Consumable income	0.004	0.003	−0.011	−0.003
	Final income	0.001	0.001	−0.007	−0.003
Poverty headcount	Disposable income			−0.022	−0.009
	Consumable income	0.009	0.005	−0.013	0.000
	Final income	0.008	0.005	−0.014	−0.003
Poverty gap	Disposable income			−0.010	−0.005
	Consumable income	0.003	0.002	−0.008	−0.002
	Final income	0.002	0.001	−0.006	−0.002
Gini	Disposable income			−0.009	−0.004
	Consumable income	−0.001	0.000	−0.010	−0.005
	Final income	−0.001	0.000	−0.009	−0.005
Budgetary savings (share of GDP):		0.014	0.007	0.000	0.008

Note

The simulation descriptions are: (1) eliminates the electricity subsidy with no compensation; (2) eliminates subsidy except for lifeline tariff for the first 50 kWh, which is held constant; (3) eliminates electricity subsidy and uses all the funds to expand LEAP, in both coverage and payments; (4) eliminates electricity subsidy and uses enough funds for LEAP to leave poverty roughly unchanged. *Source:* GLSS‐6 and authors’ calculations

The second simulation preserves the lifeline tariff rate for the first 50 kWh consumed for all consumers. This option has been proposed as a way to protect the poor from the price increases. The results suggest that this does in fact work for poverty, reducing the increase to 0.5 percentage points, but it does not help much for extreme poverty, which increases by about the same as in the first simulation. The budgetary savings are only half as much as the first simulation, but still substantial at 0.7% of GDP.

The third simulation eliminates the electricity subsidy and dedicates all the money saved to LEAP. This would be a huge increase in LEAP's budget: from 30 million (in 2013) to over a billion cedis. It is not realistic to distribute this sum to existing LEAP beneficiaries only, so we expand coverage nationwide to all people who meet the 2013 eligibility requirements.^14^ Even that does not exhaust the budgetary savings from the electricity subsidy, so we also increase the LEAP payment (24 cedis per person in 2013) by 89%. By construction, the net budgetary effect of these changes is zero, but they produce significant poverty reductions. This is because LEAP is much better targeted to the poor than are electricity subsidies. Note that disposable income changes in this simulation by the amount of additional LEAP payments only; indirect subsidies only affect consumable and final income. So the impact of expanding LEAP in the manner we suppose is to reduce poverty by 2.2 percentage points and extreme poverty by 1.3 percentage points. Including the negative effect of the subsidy removal, poverty still declines by about 1.3 percentage points, with extreme poverty a little less. Note that in this simulation, the Gini coefficient also decreases by a percentage point.

The last simulation also eliminates the electricity subsidy and expands LEAP to all eligible recipients nationwide, but in this case, only enough extra money is allocated to LEAP to keep poverty roughly constant. How much this should be varies slightly by income concept and poverty measure. We allocate enough money to LEAP to ensure that the largest poverty increase from the subsidy removal, consumable income at the national poverty line, stays at zero. This means all the other poverty measures improve slightly. The main point of this simulation is in the final row: government can keep poverty constant in the face of electricity subsidy removal by increasing expenditures on LEAP and save itself 0.8% of GDP in the process.

Table 6 gives similar results for fuel subsidies. These were about half as large in 2013 as electricity subsidies, so their impact is less, but the same broad results emerge: eliminating the fuel subsidy by itself (Simulation 1) increases poverty by small, but statistically significant amounts, while producing budgetary savings of 0.6% of GDP. If all of the budgetary savings were dedicated to expanding LEAP, poverty reduction would be a little less than 1 percentage point, with extreme poverty reduction about half that and if the government increases LEAP only just enough to offset the poverty impact of the fuel subsidy removal, the budgetary savings decline to 0.4% of GDP.

**Table 6:** Simulation Results for Eliminating Fuel Subsidies

Change in:		Simulation		
		(1)	(2)	(3)
Extreme poverty headcount	Disposable income		−0.008	−0.003
	Consumable income	0.003	−0.005	−0.001
	Final income	0.001	−0.004	−0.001
Poverty headcount	Disposable income		−0.011	−0.004
	Consumable income	0.003	−0.006	0.000
	Final income	0.003	−0.008	−0.001
Poverty gap	Disposable income		−0.005	−0.002
	Consumable income	0.001	−0.004	−0.001
	Final income	0.001	−0.003	−0.001
Gini	Disposable income		−0.004	−0.002
	Consumable income	−0.001	−0.005	−0.002
	Final income	−0.001	−0.005	−0.002
Budgetary savings (share of GDP):		0.006	0.000	0.004

Note

The simulation descriptions are: (1) eliminates the fuel subsidy with no compensation; (2) eliminates fuel subsidy and uses all the funds to expand LEAP, both coverage and payments; (3) Eliminates fuel subsidy and uses enough funds for LEAP to leave poverty roughly unchanged. *Source*: GLSS‐6 and authors’ calculations.

Overall, electricity and fuel subsidies are not effective ways to reduce poverty. The government can do better using expenditures that are well targeted to the poor, like LEAP.

### Make Taxation More Progressive?

In Ghana as in many countries, direct taxation is more progressive than indirect.^15^ Thus, the government might consider shifting from the use of indirect to direct taxation. Table 7 gives results for an extreme simulation along these lines: it eliminates the VAT and import duties, and increases the direct taxes we study here—PAYE and presumptive taxes on small enterprises—by enough to offset the revenue loss. This is clearly unrealistic as the direct tax rates would have to be extremely high, but the extremity of the example has a point: despite it, the impact on poverty and inequality is small, the largest effect being a 0.7 percentage point decline in final income poverty. Note that the poverty gaps hardly change and the change in the Gini is small.

**Table 7:** Simulation Results for Shifting from Indirect to Direct Taxation

Change in:	Extreme poverty headcount	Poverty headcount	Poverty gap	Gini
Consumable income	−0.003	−0.006	0.000	−0.003
Final income	0.000	−0.007	0.000	−0.004

Why are the effects so small? Certainly, the amounts are large: VAT plus import duties was about 6% of GDP in 2013. However, the concentration coefficients for indirect and direct taxes are not so different: 0.42 for import duties, 0.44 for VAT and 0.73 for PAYE, by far the largest source of direct taxation in this study. The difference between these is about 0.30, whereas the difference between the concentration coefficients for electricity subsidies and LEAP studied in the previous section is 0.76.

This result is important for policymakers in two ways. First, broad‐based indirect taxes like the VAT are generally considered to be more efficient than direct taxes, while direct taxes are more equitable. Thus, there is a trade‐off between equity and efficiency when choosing tax instruments,^16^ but the results here suggest that the trade‐off is not too severe. The government of Ghana can continue to rely on VAT, knowing that its use instead of direct taxation has only a minor effect on poverty and inequality. Second, the result suggests that to have a large redistributional impact, government needs to consider combinations of taxes with large positive concentration coefficients and expenditures with large negative concentration coefficients such as LEAP and school meals that are explicitly targeted to the poor.

### Change LEAP

We next consider options to expand LEAP, Ghana's conditional cash transfer (CCT) program. We should note that LEAP is not the largest, nor the best‐targeted social protection program in our study, but other alternatives would not make sense. The school‐feeding program could not expand hugely, for example, even if it were to provide quite luxurious meals.

Table 8 gives the results for five simulated changes in the LEAP program. All but the last of the changes increase LEAP expenditures to 0.5% of GDP,^17^ an amount that is typical in middle‐income countries with long established CCTs. Each simulation also pays for this increase with a VAT increase of a similar amount.

**Table 8:** Results of Simulated Changes to LEAP

*Change in:*		*Simulation*				
		*(1)*	*(2)*	*(3)*	*(4)*	*(5)*
Extreme poverty headcount	Disposable income	−0.007	−0.017	−0.019	−0.007	−0.002
	Consumable income	−0.004	−0.015	−0.017	−0.005	0.000
	Final income	−0.003	−0.012	−0.013	−0.005	0.000
Poverty headcount	Disposable income	−0.009	−0.016	−0.012	−0.008	0.000
	Consumable income	−0.004	−0.011	−0.009	−0.005	0.000
	Final income	−0.006	−0.014	−0.014	−0.006	0.000
Poverty gap	Disposable income	−0.004	−0.012	−0.012	−0.005	0.000
	Consumable income	−0.003	−0.012	−0.011	−0.004	0.000
	Final income	−0.002	−0.009	−0.009	−0.003	0.000
Gini	Disposable income	−0.004	−0.008	−0.008	−0.004	0.000
	Consumable income	−0.004	−0.009	−0.009	−0.004	0.000
	Final income	−0.003	−0.008	−0.007	−0.004	0.000
Note: Scaling factor		0.70	0.51	1.00	16.29	1.00

Note

The simulation descriptions are: (1) expands LEAP program to all eligible persons in the entire country using the old proxy means test, then scales benefits down so the total LEAP expenditure is 0.5% of GDP; (2) expands LEAP program to all people judged to be extremely poor using the new proxy means test, then scales benefits down so the total LEAP expenditure is 0.5% of GDP; (3) expands LEAP program to the poorest people as judged by the new proxy means test at current benefit rates until total LEAP payments are 0.5% of GDP; (4) increases benefits to current beneficiaries only until total LEAP payments are 0.5% of GDP; (5) keeps LEAP program payments constant, but changes to the new proxy means test. In all simulations except (5), VAT is increased to pay for the increased program size. *Source:* GLSS‐6 and authors’ calculations.

The first simulation expands LEAP to all eligible persons in the entire country using the old proxy means test: a complete expansion of the existing program. To keep the total cost to 0.5% of GDP, this requires scaling down the benefit to each recipient by 30%.

The second simulation changes the targeting to the new proxy means tests, allocating LEAP to all people judged to be extremely poor with that test. This greatly improves the targeting of LEAP, from a concentration coefficient of –0.29 to –0.65, better than most middle‐income countries.^18^ For this simulation, LEAP is given to everyone who is extremely poor, not just the elderly, handicapped and vulnerable children targeted in 2013. To keep the total cost to 0.5% of GDP, this requires scaling down the benefit to each recipient by 49% in this simulation.

The third simulation targets LEAP to the poorest people, as judged by the new proxy means test at 2013 benefit rates (no scaling down), until total LEAP payments are 0.5% of GDP. This in some sense is perfect targeting: the money goes to the poorest people in the sample.

The fourth simulation increases benefits to 2013 beneficiaries only until total LEAP payments reach 0.5% of GDP—that is, it uses the current targeting only. Because current beneficiaries are so few, this produces a huge and unrealistic payment to them: 16 times larger than the current 24 cedis per person per month.

The fifth simulation keeps the program size constant at the 2013 level of 0.02% of GDP, much smaller than the other simulations, and changes the targeting to the new proxy means test.

To interpret the results, recall that disposable income comes prior to VAT, so the impact shown for disposable income is the impact of the LEAP increase only, while impacts for consumable and final income account for both the additional LEAP and its assumed financing via additional VAT.

The first simulation shows that increasing LEAP to nationwide coverage using existing targeting accuracy while holding the overall budget to 0.5% of GDP would reduce disposable income poverty by 0.9 percentage points and extreme poverty by 0.7. Including the effect of the VAT increase reduces the gains, to between 0.3 and 0.6 percentage points, depending on the poverty line and income concept. Reductions in the Gini are small: 0.4 percentage points.

The second simulation does much better, demonstrating the advantages of better targeting. Here, disposable income poverty declines by 1.6 percentage points and extreme poverty by 1.7. Including the losses from imposing additional VAT, the gains are still much larger: between 1.1 and 1.5 percentage points. The improvements in the poverty gap are even stronger, reflecting the fact that some of the poorest recipients of LEAP will not cross the poverty line even with its assistance, but their poverty gap will decrease nonetheless.

The third simulation is “perfect targeting,” but it does just about as well as the second. In fact, it does a little worse on some of the measures. How can this be? Here, LEAP is perfectly targeted to the PMT value, but not the actual incomes used to calculate the poverty rates, and the rank correlation of the PMT and incomes is not perfect. The key conclusion from this simulation, though, is more that using the targeting of the second simulation is about as good as can be achieved with the new PMT.

Results for the fourth simulation are very similar to the first, as both use the old PMT. It is interesting, though, to note that the poverty and inequality effects of an expansion of LEAP's extensive margin (adding new beneficiaries) and intensive margin (increasing benefits to existing beneficiaries) are broadly similar.

Finally, the fifth simulation shows almost no change in poverty or inequality measures, despite the switch to the better targeting of the new PMT, because the program size does not change here. Thus, even greatly improved targeting of a small program cannot have much impact on poverty and inequality. Larger program size is essential.

## 5 Conclusions

The analysis began with three questions about the redistributive effect of taxes and expenditures:

1. How much redistribution and income poverty reduction is being accomplished through social spending, subsidies and taxes?
2. How progressive are revenue collection, subsidies and government social spending?
3. Within the limits of fiscal prudence, what could be done to increase redistribution and poverty reduction through changes in taxation and spending?

The answer to the first question is: with the exception of public spending on health care and (especially) education, not much. Overall, the government spending and taxation analyzed here, which we should remember are only a fraction of all spending and taxation, reduce the Gini coefficient by 0.035, which is at the low end of the mostly middle‐income countries analyzed by CEQ. Results for poverty reduction are even less encouraging. Were it not for the in‐kind benefits from health and education spending, the fisc would actually increase poverty in Ghana, by about two points. This is almost entirely because poor people pay indirect taxes in Ghana, as in every other country.

For the second question, Ghana certainly imposes some highly progressive taxes including PAYE and most excises. The main indirect taxes, VAT and import duties, are distributionally neutral, while the cocoa duty stands out as a regressive tax.

On the expenditure side, Ghana has some moderately well‐targeted expenditures. Some of these such as LEAP and the school‐feeding program are explicitly targeted to the poor, while others such as schooling through junior high school should be universal, but achieve better targeting as wealthier households self‐select out of public schools.

There are also some very poorly targeted expenditures in Ghana, most notably subsidies to electricity, which were substantial in 2013, and subsidies to petrol and diesel that are necessitated by holding down the retail price. Subsidies to higher education, especially university education, are also captured by richer households.

Within the limits of fiscal prudence, what could be done to increase redistribution and poverty reduction through changes in taxation and spending? Most importantly, the government's move to eliminate all subsidization of fuel and electricity is welcome. These are regressive subsidies and, at times, are quite costly to the fisc. It is true that elimination of these subsidies will increase poverty, but it is also true that, if poverty reduction is the main goal, government could achieve a similar level of poverty reduction with much less expenditure by focusing on its well‐targeted programs rather than energy subsidies.

While it may seem odd to argue for tax reductions while Ghana faces large fiscal deficits, there is a strong case for eliminating the cocoa duty. This is a regressive tax and it is also inefficient, discouraging production of a key export product.

Within the education sector, our results are similar to prior results in Ghana and everywhere else in the world: lower levels are more progressive than higher. So reallocation of subsidies from post‐secondary to primary would be progressive. For health, however, Ghana does not show the typical pattern of outpatient care being more progressive than inpatient. Finally, education and health expenditures are the main way that the budget reduces poverty in Ghana, not because they have the best targeting, but because they have large budgets.

Are there lessons in Ghana's experience for other countries? We think there are three key ones. First, Ghana has shown that a lower‐middle income country can target social expenditures extremely well if it decides to do so. This is the case for both LEAP and the school meals program. Second, good targeting is necessary for substantial poverty reduction, but not sufficient. Programs also need to have large budgets. Thus the biggest poverty reduction in Ghana comes from education and health spending, not because they have the best targeting, but because they have large budgets. Finally, the targeting of these programs is far better than other ostensibly pro‐poor policies, most notably subsidies to petroleum products and electricity, neither of which is progressive in Ghana, nor in many other countries (International Monetary Fund (IMF), 2015).

## Footnotes

1 For details see http://www.commitmentoequity.org.

2 In particular, we use the Gini coefficient and Foster–Greer–Thorbecke (FGT) poverty measures. For the latter, we consider both the national poverty line established by the Ghana Statistical Service to allow comparison of our results with national publications, and international “dollar‐a‐day” poverty lines to allow comparisons to other countries.

3 A working paper version of this paper provides greater detail on the construction of the income concepts. See Younger et al. (2016).

4 It seems likely that this discrepancy occurs because the GLSS asks about hospitalizations in the past two weeks only, but respondents may be reporting them for a much longer period, “telescoping” those visits into the reference period.

5 These latter revenues include internally generated funds at public schools and health providers.

6 The results presented here focus only on our own work using GLSS‐6. Younger et al. (2016) compare these results with previous incidence studies done in Ghana and similar CEQ studies in other countries.

7 Because our “disposable income” is identical to GSS's household consumption, the poverty values for disposable income in Table 3 are identical to the national poverty measures in Ghana.

8 To be precise, the ones measured here: PAYE and presumptive taxes.

9 The exception is at the highest poverty line (US$4.00 per day) where direct taxes increase poverty by about 1%.

10 It is also the case that if we assume that only 50% of the incidence of indirect taxes is borne by consumers, these results, while qualitatively similar, are much smaller in magnitude. See Younger et al. (2016, appendix III) for a discussion.

11 Enami et.al. (2017) show that this statement is not strictly true if the tax or benefit generates a significant re‐ranking of people in the income distribution. They give examples of transfers to the poorest that are large enough to move them well up the income distribution and show that these transfers reduce the Gini less than similarly sized transfers spread more evenly across the population. Nevertheless, the size of taxes and transfers in Ghana are such that the intuition of the text is adequate.

12 Clementi et al. (2015) make a similar calculation for the elimination of fuel subsidies and find that the cost of offsetting the poverty impact via an expansion of LEAP would be less than 50% of the expenditure on fuel subsidies.

13 This is larger than the 1.25% reported above, because we use the estimate of the subsidy from the GLSS, which is slightly larger than the amount in Ministry of Finance accounts.

14 We use the older proxy means test when determining eligibility. The Ministry of Gender, Children and Social Protection has changed the proxy means test using GLSS‐6 data, an effort that may improve LEAP's targeting and make results even better than what we simulate here.

15 The exception is for some of the excise taxes.

16 Administrative efficiency should also enter the policy mix.

17 At the time of the GLSS‐6 survey, LEAP was still a quite small program, limited to only select districts in the country. Government has gradually expanded coverage since then.

18 In practice, the new PMT will not work this well. Because it is estimated using the same GLSS‐6 data that we use here, it is particularly well suited to identifying the poor in this sample, but because of measurement error, will do less well in the general population.
